# AHR Signaling Interacting with Nutritional Factors Regulating the Expression of Markers in Vascular Inflammation and Atherogenesis

**DOI:** 10.3390/ijms21218287

**Published:** 2020-11-05

**Authors:** Carla Dahlem, Sarah Y. Kado, Yi He, Keith Bein, Dalei Wu, Thomas Haarmann-Stemmann, Norman Y. Kado, Christoph F. A. Vogel

**Affiliations:** 1 Center for Health and the Environment, University of California, One Shields Avenue, Davis, CA 95616, USA; carla.dahlem@uni-konstanz.de (C.D.); sykado@ucdavis.edu (S.Y.K.); fyhe@ucdavis.edu (Y.H.); kjbein@ucdavis.edu (K.B.); dlwu@sdu.edu.cn (D.W.); 2 University Konstanz, Universitätsstraße 10, 78464 Konstanz, Germany; 3 Helmholtz International Lab, State Key Laboratory of Microbial Technology, Shandong University, Qingdao 266237, China; 4 Leibniz Research Institute for Environmental Medicine, 40225 Düsseldorf, Germany; Thomas.Haarmann-Stemmann@IUF-Duesseldorf.de; 5 Department of Environmental Toxicology, University of California, One Shields Avenue, Davis, CA 95616, USA; nykado@ucdavis.edu

**Keywords:** AHR, atherosclerosis, cytokines, inflammation, macrophages, obesity, PM, TCDD

## Abstract

There is strong evidence that exposure to fine particulate matter (PM_2.5_) and a high-fat diet (HFD) increase the risk of mortality from atherosclerotic cardiovascular diseases. Recent studies indicate that PM_2.5_ generated by combustion activates the Aryl Hydrocarbon Receptor (AHR) and inflammatory cytokines contributing to PM_2.5_-mediated atherogenesis. Here we investigate the effects of components of a HFD on PM-mediated activation of AHR in macrophages. Cells were treated with components of a HFD and AHR-activating PM and the expression of biomarkers of vascular inflammation was analyzed. The results show that glucose and triglyceride increase AHR-activity and PM_2.5_-mediated induction of cytochrome P450 (CYP)1A1 mRNA in macrophages. Cholesterol, fructose, and palmitic acid increased the PM- and AHR-mediated induction of proinflammatory cytokines in macrophages. Treatment with palmitic acid significantly increased the expression of inflammatory cytokines and markers of vascular injury in human aortic endothelial cells (HAEC) after treatment with PM_2.5_. The PM_2.5_-mediated activation of the atherogenic markers C-reactive protein (CRP) and S100A9, a damage-associated molecular pattern molecule, was found to be AHR-dependent and involved protein kinase A (PKA) and a CCAAT/enhancer-binding protein (C/EBP) binding element. This study identified nutritional factors interacting with AHR signaling and contributing to PM_2.5_-induced markers of atherogenesis and future cardiovascular risk.

## 1. Introduction

The Aryl Hydrocarbon Receptor (AHR) is known to mediate the toxicity of environmental pollutants such as dioxins and dioxin-like compounds. The dioxin congener 2,3,7,8-tetrachlorodibenzo-p-dioxin (TCDD) is the most potent environmental toxicant and has been used in numerous studies as a prototypical ligand to activate AHR [1]. In recent years, air pollution and particulate matter (PM) generated by combustion have been found to contain significant amounts of polycyclic aromatic hydrocarbons (PAHs), another class of agonists, which can activate the AHR signaling pathway [2]. Furthermore, there is strong evidence that exposure to fine particulate matter (PM_2.5_) and PAHs contribute to the incidence of atherosclerosis and cardiovascular diseases (CVD) [3,4]. Atherosclerosis is a chronic inflammatory condition and the primary cause of ischemic heart disease and stroke, which is linked to about 50% of all deaths in Western countries [5,6].

AHR-dependent activation of cytochrome P450 (CYP) 1A1 and inflammatory cytokines such as interleukin (IL)-8 and IL-1β after exposure to PM have been shown in different cell types as well as in in vivo [7,8,9,10,11,12,13]. Recent studies indicate that PAHs are major contributors to PM-mediated atherogenesis and AHR-induced toxicity [14,15,16,17,18,19]. The AHR and its DNA binding partner, the AHR nuclear translocator (ARNT), are central in the regulation of CYP1 enzymes and mediating PAH-induced gene expression [1]. Recent studies including our work implicate the interaction of PM_2.5_ collected in an urban area (Sacramento, CA) with AHR as a key event leading to elevated levels of pro-inflammatory cytokines, such as IL-1β, IL-8, C-C motif chemokine ligand (CCL) and C-X-C motif chemokine ligand (CXCL) chemokines associated with the promotion of Th17-immune responses [7,20,21]. Furthermore, we showed that activation of AHR plays an important role in foam cell formation and accumulation of lipids [22]. The AHR-mediated foam cell formation was associated with increased levels of IL-1β in atherosclerotic plaques [23]. Besides inflammatory cytokines, C-reactive protein (CRP) and S100A9, a damage-associated molecular pattern molecule, have been identified as critical biomarkers in vascular inflammation and plaque disruption [24,25].

Despite air pollutants and PM, obesity is known as a significant risk factor for the development of chronic inflammatory diseases such as atherosclerosis [6]. Recent studies suggest that obesity factors present in HFD may initiate an inflammatory response [26]. For instance, cholesterol (Chol), elevated levels of glucose (Gluc) and triglyceride (TGL), and saturated fatty acids such as palmitic acid (Palm) are known to have pro-inflammatory effects and participate in the development of atherosclerosis [27,28,29,30]. Given the high prevalence of obesity and AHR-active air pollution worldwide, and its association with CVD understanding the role of AHR in inflammation-mediated atherogenesis could have a strong impact on the prevention and treatment of atherosclerosis. In the current study we examined the interaction of the AHR activated by exposure to PM_2.5_ combined with components of a HFD on the expression of markers of vascular inflammation and atherogenesis in macrophages and human aortic endothelial cells (HAEC).

## 2. Results

To investigate the role of AHR activation in cardiovascular diseases an in vitro setup with human U937-derived macrophages (Umac) and HAEC was established. The cells were treated for 24 h with the potent AHR ligand TCDD or with PM_2.5_ collected from a major freeway tunnel system in Northern California as described under Materials and Methods. Cells were cotreated with nutritional factors of a high fat diet to investigate their effects on the induction of AHR target genes. 

### 2.1. CYP1A1 Expression in Response to PM_2.5_ and Nutritional Factors

**Figure 1:**
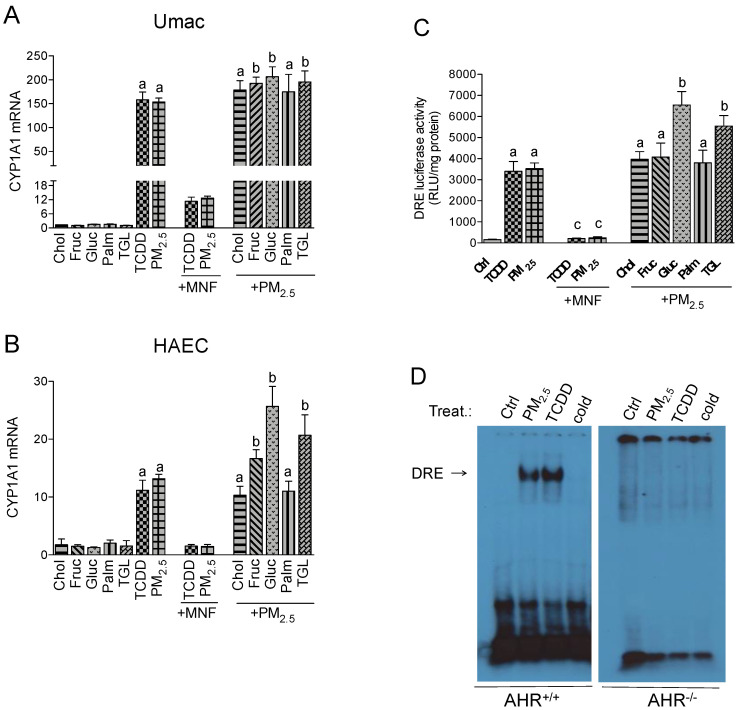
Effect of nutritional compounds on tetrachlorodibenzo-p-dioxin (TCDD)- and particulate matter (PM_2.5_)-induced cytochrome P450 (CYP)1A1 expression and Aryl Hydrocarbon Receptor (AHR) activity. (**A**) U937-derived macrophages (Umac) and (**B**) human aortic endothelial cells (HAEC) were treated with cholesterol (Chol, 10 μg/mL), fructose (Fruc, 25 mM), glucose (Gluc, 25 mM), palmitic acid (Palm, 5 μM), triglyceride (TGL, 10 μg/mL), TCDD (1 nM), and PM_2.5_ (10 μg/mL) for 24 h. Cells were treated with 3-methoxy-4-nitroflavone (MNF) (5 μM) to block activation of AHR by TCDD and PM_2.5_. The effect of nutritional factors on CYP1A1 was tested in presence of PM_2.5_. The mRNA expression of CYP1A1 and the housekeeping ß-actin was analyzed by qPCR. Control cells were treated with the corresponding vehicle. (**C**) Effect of nutritional compounds on PM_2.5_-induced dioxin responsive element (DRE) luciferase activity. Umac were transiently transfected with a DRE luciferase reporter construct for 24 h and then treated with TCDD or PM_2.5_ in presence or absence of MNF for 16 h. The effect of nutritional factors on DRE activity was tested in presence of PM_2.5_; ^a^ significantly higher than Ctrl; *^b^* significantly higher than cells treated with PM_2.5_ only; *^c^* significantly lower than cells treated with TCDD or PM_2.5_ only; *p* < 0.05; (**D**) PM_2.5_-induced AHR binding activity. Bone marrow-derived macrophages (BMM) from AHR^+/+^ and AHR^−/−^ mice were treated for 1 h with 10 μg/mL of PM_2.5_, cells were also treated with 1 nM TCDD as a positive control. Nuclear proteins were extracted and incubated with ^32^P-labeled oligonucleotides containing the consensus site of DRE and loaded on a native gel for gel-mobility shift assay (GMSA). A 100-fold excess of the unlabeled specific (cold) DRE oligonucleotides was added to confirm specificity.

Focusing on the expression levels of CYP1A1 as a direct indicator of AHR activity, the single treatment with nutritional factors had no significant effect on CYP1A1 mRNA levels compared to control in Umac and HAEC (Figure 1A,B). TCDD treatment resulted in a 160- and 11-fold increase in CYP1A1 mRNA levels for Umac (Figure 1A) and HAEC (Figure 1B), respectively. Thereafter we show here that PM_2.5_ leads to a similar induction of CYP1A1 like TCDD (150- and 13-fold) indicating the potency of PM_2.5_ to activate AHR. To verify the relevance of AHR activity to induce the expression of CYP1A1, the cells were treated with the AHR antagonist 3-methoxy-4-nitroflavone (MNF). With MNF treatment the mRNA levels remained low, which indicates the dependence on AHR activation. The addition of cholesterol or palmitic acid to the PM_2.5_ treatment did not result in an additional increase in CYP1A1 mRNA levels. However, the co-treatment of PM_2.5_ with fructose, glucose or triglyceride further elevated the PM_2.5_-induced expression. This effect was most prominent in HAEC cells, when cotreated with PM_2.5_ and glucose (Figure 1B). 

### 2.2. Effect of Nutritional Factors on PM_2.5_-Mediated AHR Activity

To verify the AHR-dependent activation of PM_2.5_-induced expression of CYP1A1 and other target genes, a luciferase reporter assay was performed with Umac. TCDD and PM_2.5_ both significantly induced the dioxin responsive element (DRE)-dependent luciferase activity of AHR (Figure 1C). The AHR antagonist MNF blocked about 90% of TCDD- and PM_2.5_-induced luciferase activity confirming the AHR-dependent activation. Co-treatment with cholesterol, fructose or palmitic acid did not significantly change the effect of PM_2.5_-induced AHR activity. However, the addition of glucose and triglyceride further increased the luciferase activity 2-fold and 1.5-fold, respectively. The results agree with the effects observed by glucose and triglyceride on CYP1A1 mRNA levels. No enhanced AHR-dependent luciferase activity was found after cotreatment with cholesterol, fructose or palmitic acid (Figure 1C). As additional evidence of PM_2.5_-induced activation of AHR, a GMSA was performed with nuclear proteins from bone marrow-derived macrophages (BMM) from AHR wild type (AHR^+/+^) and AHR deficient (AHR^−/−^) mice treated with PM_2.5_ for 1 h. TCDD was used as a positive control. In BMM derived from mice expressing the AHR, TCDD and PM_2.5_ treatment significantly increased the DRE binding activity (Figure 1D). No significant DRE binding activity was detected in PM_2.5_- or TCDD-treated BMM from AHR^−/−^ mice. 

### 2.3. PM_2.5_ and Nutritional Factor Induced Expression of Pro-Inflammatory Cytokines

**Figure 2:**
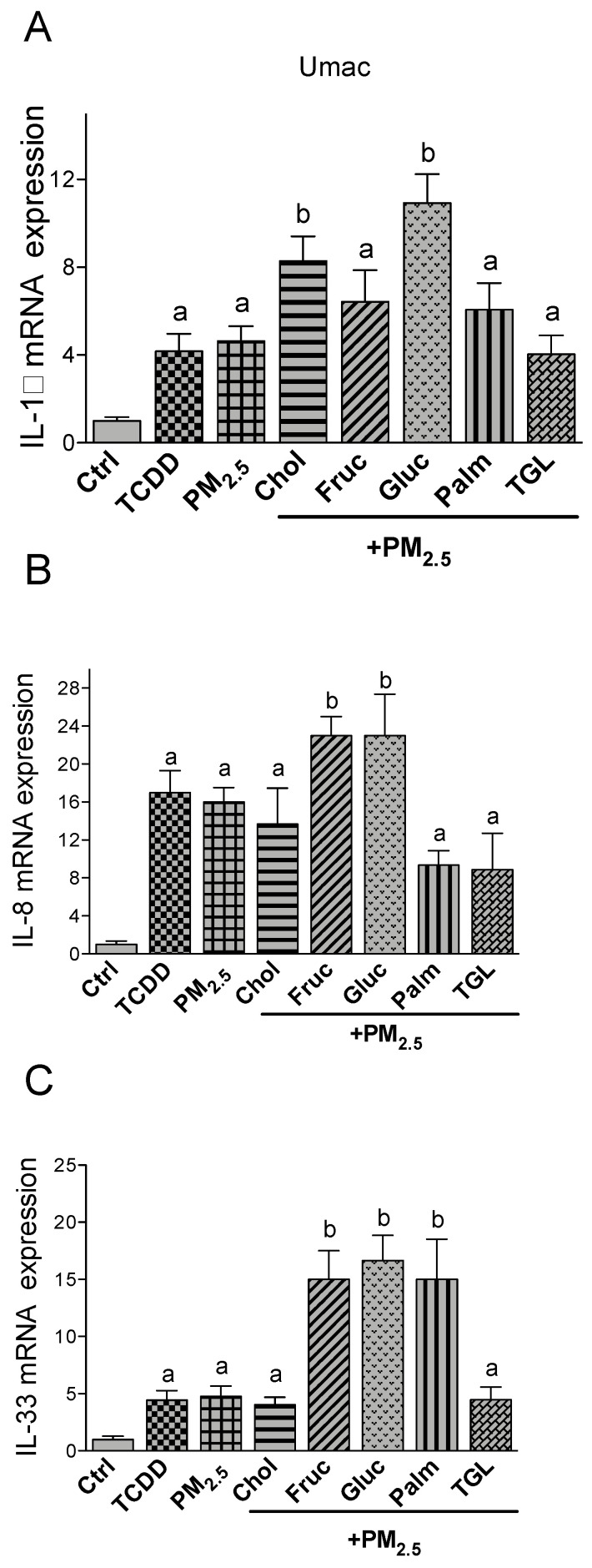
Effect of nutritional compounds on PM_2.5_-induced cytokine expression in Umac. Cells were treated with 1 nM TCDD and 10 μg/mL PM_2.5_ for 24 h. The effect of nutritional factors cholesterol (Chol, 10 μg/mL), fructose (Fruc, 25 mM), glucose (Gluc, 25 mM), palmitic acid (Palm, 5 μM), and triglyceride (TGL, 10 μg/mL) was tested in presence of PM_2.5_ after 24 h treatment. Control cells were treated with the corresponding vehicle. The mRNA expression of (**A**) IL-1β; (**B**) IL-8, and (**C**) IL-33 was analyzed by qPCR. The expression was corrected against the housekeeping gene ß-actin. ^a^ significantly higher than Ctrl; ^b^ significantly higher than cells treated with PM_2.5_ only *p* < 0.05.

**Figure 3:**
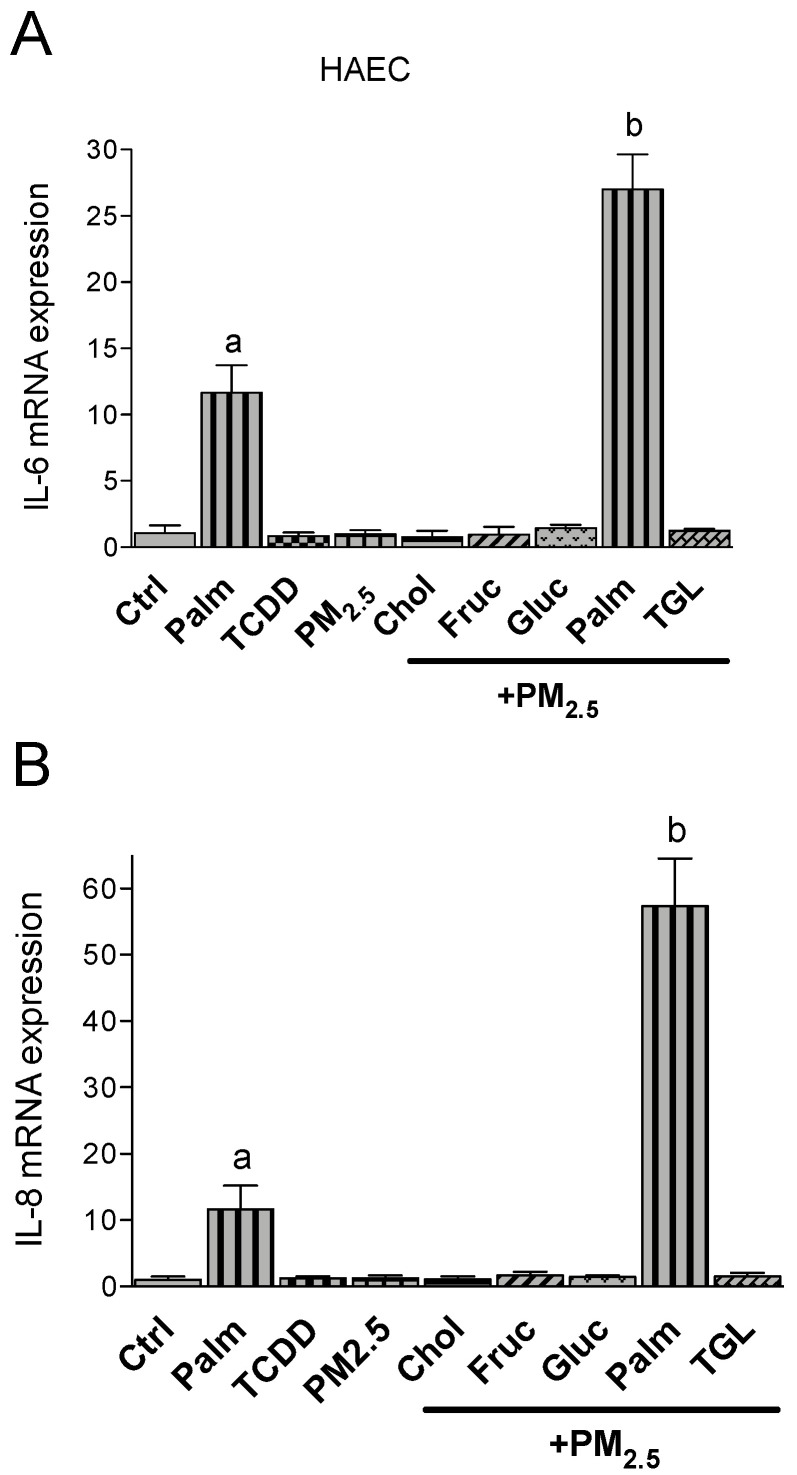
Effect of nutritional compounds on PM_2.5_-induced cytokine expression in HAEC. Cells were treated with 1 nM TCDD and 10 μg/mL PM_2.5_ for 24 h. The effect of nutritional factors cholesterol (Chol, 10 μg/mL), fructose (Fruc, 25 mM), glucose (Gluc, 25 mM), palmitic acid (Palm, 5 μM), and triglyceride (TGL, 10 μg/mL) was tested in presence of PM_2.5_ after 24 h of treatment. Control cells were treated with the corresponding vehicle. The mRNA expression of (**A**) IL-6 and (**B**) IL-8 was analyzed by qPCR. The expression was corrected against the housekeeping gene β-actin. ^a^ significantly higher than Ctrl; ^b^ significantly higher than cells treated with PM_2.5_ only *p* < 0.05.

In Umac IL-1β, IL-8 and IL-33 were induced after TCDD or PM_2.5_ treatment by 4-, 17- and 5-fold over control levels, respectively (Figure 2). No significant differences were observed between TCDD and or PM_2.5_. The treatment with nutritional factors alone had no significant effect on IL-1β, IL-8 or IL-33 mRNA levels compared to the control (data not shown). The treatment with cholesterol and glucose together with PM_2.5_ further increased the mRNA level of IL-1β by 2- or 2.5-fold, respectively. Fructose, triglyceride and palmitic acid did not affect the expression levels of PM_2.5_-induced IL-1β (Figure 2A). The expression of IL-8 was further elevated by the treatment of PM_2.5_ in the presence of glucose and fructose. Cholesterol, palmitic acid and triglyceride co-treatment resulted in a less pronounced induction of IL-8 compared to PM_2.5_ only (Figure 2B). Macrophages treated with PM_2.5_ in combination with palmitic acid as well as glucose and fructose showed a significant upregulation of IL-33 expression compared to PM_2.5_ only (3-fold). Additional treatment with cholesterol or triglyceride had no significant effect on IL-33 in Umac (Figure 2C). Furthermore, aortic endothelial cells are an important target cell in CVD and the expression of pro-inflammatory cytokines are involved in the formation of arthrosclerosis. With the exception of palmitic acid none of the nutritional factors had a significant effect on the expression of IL-6 or IL-8 (Figure 3A,B) in HAEC. Single treatment with TCDD or PM_2.5_ alone had no effect on IL-6 or IL-8 expression in HAEC. The upregulation of IL-6 and IL-8 by palmitic acid was further increased by co-treatment with PM_2.5_. PM_2.5_ enhanced the palmitic acid-mediated induction of IL-6 by 3-fold and by 6-fold for IL-8. 

### 2.4. PM_2.5_ and Nutritional Factor Induced Expression of Atherogenic Markers

**Figure 4:**
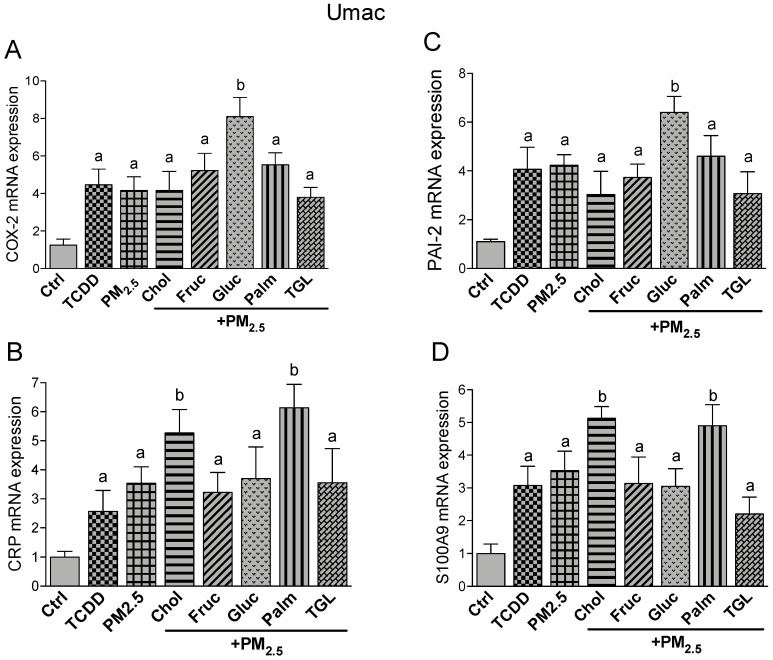
Effect of nutritional compounds on PM_2.5_-induced expression of atherogenic markers in Umac; Cells were treated with 1 nM TCDD and 10 μg/mL PM_2.5_ for 24 h. The effect of nutritional factors cholesterol (Chol, 10 μg/mL), fructose (Fruc, 25 mM), glucose (Gluc, 25 mM), palmitic acid (Palm, 5 μM), and triglyceride (TGL, 10 μg/mL) was tested in presence of PM_2.5_ after 24 h treatment. Control cells were treated with the corresponding vehicle. The mRNA expression of (**A**) COX-2; (**B**) CRP; (**C**) PAI-2, and (**D**) S100A9 was analyzed by qPCR. The expression was corrected against the housekeeping gene β-actin. ^a^ significantly higher than Ctrl; ^b^ significantly higher than cells treated with PM_2.5_ only *p* < 0.05.

Besides pro-inflammatory cytokines, the activated AHR acts as transcription factor and affects several other target genes which are critically involved in atherogenesis. AHR-mediated and CVD related factors like cyclooxygenase (COX)-2, CRP, Plasminogen activator inhibitor 2 (PAI-2) and the damage-associated molecular pattern molecule S100A9 were investigated in Umac. As for the cytokines, treatment with nutritional factors alone had no significant effect on COX-2, CRP, PAI-2 and S100A9 mRNA levels compared to the control (data not shown). The expression of COX-2 was equally induced by TCDD and PM_2.5_ and further upregulated by additional treatment with glucose (Figure 4A). A similar expression pattern was found for PAI-2 (Figure 4C). Cholesterol, fructose, palmitic acid or triglyceride co-treatment was not sufficient to further increase the expression of PM_2.5_-induced COX-2 or PAI-2 (Figure 4A,C). While the addition of glucose resulted in a significant upregulation of COX-2 and PAI-2, glucose treatment did not lead to a significant change in PM_2.5_-induced expression of CRP or S100A9 (Figure 4B,D). The expression of CRP and S100A9 was induced by treatment with TCDD and PM_2.5_. Cotreatment with cholesterol or palmitic acid increased the mRNA levels of CRP as well as S100A9 induced by PM_2.5_.

**Figure 5:**
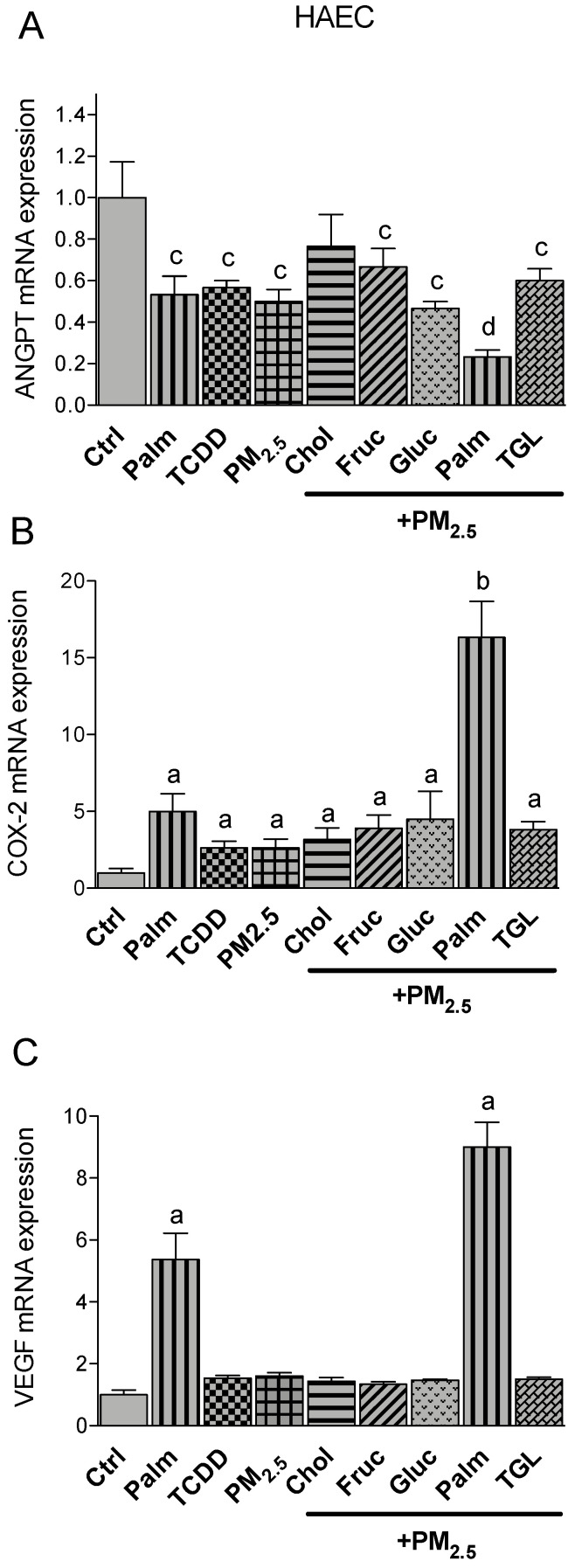
Effect of nutritional compounds on PM_2.5_-induced expression of atherogenic markers in HAEC; Cells were treated with 1 nM TCDD and 10 μg/mL PM_2.5_ for 24 h. The effect of nutritional factors cholesterol (Chol, 10 μg/mL), fructose (Fruc, 25 mM), glucose (Gluc, 25 mM), palmitic acid (Palm, 5 μM), and triglyceride (TGL, 10 μg/mL) was tested in presence of PM_2.5_ after 24 h treatment. Control cells were treated with the corresponding vehicle. The mRNA expression of (**A**) Angiopoetin (ANGPT), (**B**) COX-2, and (**C**) VEGF was analyzed by qPCR. The expression was corrected against the housekeeping gene ß-actin. ^a^ significantly higher than Ctrl; ^b^ significantly higher than cells treated with PM_2.5_ or Palm only; ^c^ significantly lower than Ctrl; ^d^ significantly lower than cells treated with Palm or PM_2.5_ only *p* < 0.05.

In the human endothelial cells HAEC we analyzed the expression of angiopoietin (ANGPT), COX-2 and vascular endothelial growth factor (VEGF). Analysis of the expression levels of ANGPT in HAEC showed that treatment with palmitic acid, TCDD and PM_2.5_ decreased the expression of ANGPT. Co-treatment with palmitic acid and PM_2.5_ further reduced the expression of ANGPT by about 70% of the control level (Figure 5A). On the other hand, similar to proinflammatory cytokines, the expression of COX-2 and VEGF was significantly upregulated (5-fold) by palmitic acid (Figure 5B,C). The combination of PM_2.5_ with palmitic acid further increased the expression of COX-2 and VEGF up to 15-fold and 9-fold, respectively, above control. No effect was observed with PM_2.5_ cotreated with the other obesity factors tested.

### 2.5. Effect of Nutritional Factors on PM_2.5_-Mediated CRP and S100A9 Promoter Activity

**Figure 6:**
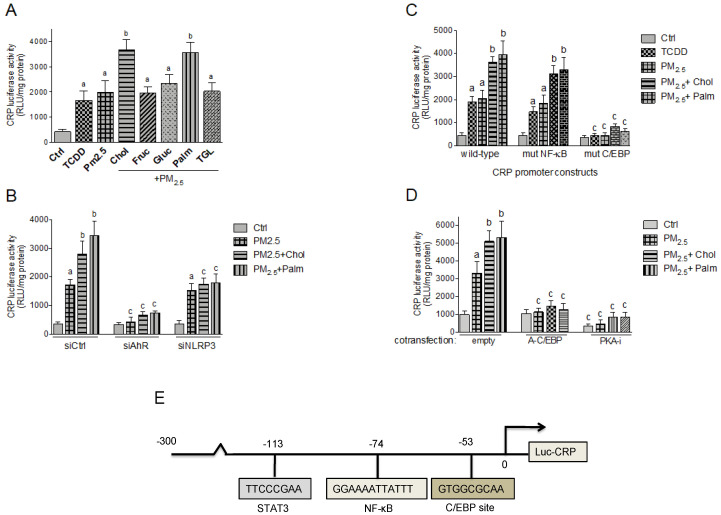
Effect of nutritional compounds on PM_2.5_-induced CRP promoter activity. (**A**) Umac were transiently transfected with a luciferase promoter construct containing 300 bp upstream of the regulatory sequence of the human CRP gene promoter. After 24 h cells were treated with 1 nM TCDD and 10 μg/mL PM_2.5_ in presence or absence of nutritional factors cholesterol (Chol, 10 μg/mL), fructose (Fruc, 25 mM), glucose (Gluc, 25 mM), palmitic acid (Palm, 5 μM), and triglyceride (TGL, 10 μg/mL) for 6 h. Control cells were treated with the corresponding vehicle only. (**B**) Umac were transiently transfected with the CRP wt luciferase promoter construct and co-transfected with a scrambled control siRNA, a AHR-specific or NLRP3-specific siRNA. After 24 h cells were treated with 10 μg/mL PM_2.5_ in the presence or absence of cholesterol (Chol, 10 μg/mL) or palmitic acid (Palm, 5 μM). (**C**) Umac were transiently transfected with the luciferase reporter constructs of the wt CRP promoter or a CRP construct containing a mutation in the NF-kB (mut NF-kB) or C/EBP binding site (mut C/EBP). After 24 h cells were treated with 1 nM TCDD or 10 μg/mL PM_2.5_ in the presence or absence of cholesterol (Chol, 10 μg/mL) or palmitic acid (Palm, 5 μM). (**D**) PM_2.5_-induced CRP promoter activity is C/EBP- and PKA-dependent. Cells were co-transfected with an empty, C/EBP-A dominant negative expression plasmid, or a PKA inhibitor (PKA-i) expression plasmid for 24 h and treated with 10 μg/mL PM_2.5_ in presence or absence of cholesterol (Chol, 10 μg/mL) or palmitic acid (Palm, 5 μM) for 6h. Relative luciferase activity units (RLU) are given as mean values of triplicates as a result of three independent experiments. ^a^ significantly higher than control (*p* < 0.05); ^b^ significantly higher than cells treated with PM_2.5_ only (*p* < 0.05); ^c^ significantly lower than cells transfected with ctrl siRNA, wild-type CRP, or an empty vector *(p* < 0.05). (**E**) Schematic illustration of the promoter construct of the human CRP gene containing 300 bp upstream of the transcriptional start site (indicated by an arrow) cloned into a luciferase (luc) reporter vector. Positions of the C/EBP, NF-κB, and STAT3 recognition sites are presented.

**Figure 7:**
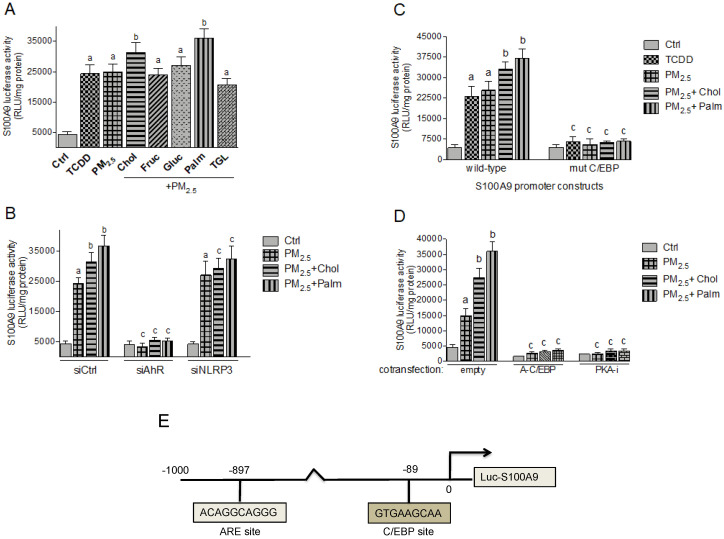
Effect of nutritional compounds on PM_2.5_-induced S100A9 promoter activity. (**A**) Umac were transiently transfected with a luciferase promoter construct containing 1000 bp upstream of the regulatory sequence of the human S100A9 gene promoter. After 24 h cells were treated with 1 nM TCDD and 10 μg/mL PM_2.5_ in the presence or absence of nutritional factors cholesterol (Chol, 10 μg/mL), fructose (Fruc, 25 mM), glucose (Gluc, 25 mM), palmitic acid (Palm, 5 μM), and triglyceride (TGL, 10 μg/mL) for 6 h. Control cells were treated with the corresponding vehicle. (**B**) Umac were transiently transfected with the S100A9 wt luciferase promoter construct and co-transfected with a scrambled control siRNA, a AHR-specific or NLRP3-specific siRNA. After 24 h cells were treated with 10 μg/mL PM_2.5_ in the presence or absence of cholesterol (Chol, 10 μg/mL) or palmitic acid (Palm, 5 μM). (**C**) Umac were transiently transfected with the luciferase reporter constructs of the wt S100A9 promoter or a construct containing a mutation in the C/EBP binding site. After 24 h cells were treated with 1 nM TCDD or 10 μg/mL PM_2.5_ in the presence or absence of cholesterol (Chol, 10 μg/mL) or palmitic acid (Palm, 5 μM). (**D**) Cells were co-transfected with an empty, C/EBP-A dominant negative expression plasmid, or a PKA inhibitor (PKA-i) expression plasmid for 24 h and treated with 10 μg/mL PM_2.5_ for 6h in the presence or absence of cholesterol (Chol, 10 μg/mL) or palmitic acid (Palm, 5 μM). Relative luciferase activity units (RLU) are given as mean values of triplicates as a result of three independent experiments. ^a^ significantly higher than control (*p* < 0.05); ^b^ significantly higher than cells treated with PM_2.5_ only (*p* < 0.05); ^c^ significantly lower than cells transfected with ctrl siRNA, wild-type S100A9, or an empty vector *(p* < 0.05). (**E**) Schematic illustration of promoter construct of the human S100A9 gene containing 1000 bp upstream of the transcriptional start site (indicated by an arrow) cloned into a luciferase (luc) reporter vector. Positions of the C/EBP recognition site and an antioxidant response element (ARE) are presented.

The damage-associated molecular pattern molecule CRP and S100A9 are important factors in the inflammatory process of atherosclerosis. To test the effect of PM_2.5_ and nutritional factors on transcriptional activities of CRP and S100A9 we used luciferase reporter constructs containing 300 bp and 1000 bp of the regulatory sequences upstream of the start sites from the human CRP and S100A9 genes, respectively. Here the luciferase activity is under control of the CRP and S100A9 promotor. Treatment with TCDD and PM_2.5_ induced the activity of CRP and S100A9 (Figure 6A and Figure 7A). The combination of PM_2.5_ with cholesterol or palmitic acid further increased the promoter activity of CRP and S100A9.

To test whether PM_2.5_-induced activity of CRP and S100A9 is AHR-dependent, Umac were co-transfected with AHR specific silencing RNA. As shown in Figure 6B and Figure 7B, siAHR led to a clear inhibition of PM_2.5_ mediated luciferase activity of CRP and S100A9. Co-transfecting a silencing RNA specific for human NLRP3 did not change the PM_2.5_ -induced activity, but suppressed the additional effect by cholesterol and palmitic acid on S100A9 and CRP activity. The sequences of the promoter constructs of CRP and S100A9 used in this study do not contain a consensus DRE binding site (Figure 6E and Figure 7E). Therefore, we investigated other potential signaling pathways and binding elements involved in the regulation of CRP and S100A9. A promoter construct containing the mutated NF-kB binding site at position -74 bp of the CRP promoter did not significantly change the TCDD- and PM_2.5_-induced activity (Figure 6C). However, the mutation of the C/EBP binding sites located at position -53 bp of the CRP and at -89 bp of the S100A9 promoter sequence suppressed the TCDD- and PM_2.5_-induced activity (Figure 6C and Figure 7C). Furthermore, co-transfection studies with a PKA inhibitor (PKA-i) and C/EBP dominant negative expression plasmid (A-C/EBP) completely blocked the TCDD- and PM_2.5_-induced activity of CRP and S100A9 (Figure 6D and Figure 7D).

## 3. Discussion

**Figure 8:**
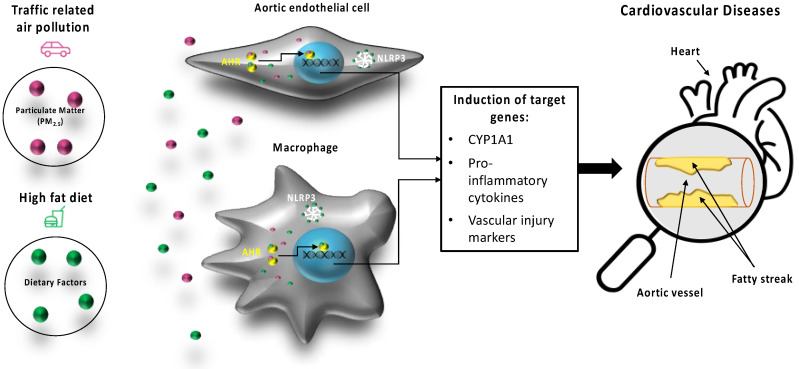
Effects of PM_2.5_ derived from traffic related air pollution (TRAP) and dietary factors of a high fat diet (HFD) on target cells of the cardiovascular system such as aortic endothelial cells and macrophages. AHR and NLRP3 mediate the activation of atherogenic markers and cardiovascular diseases induced by PM_2.5_ and HFD. The earliest visible lesion of atherosclerosis is the fatty streak as indicated by arrows. The fatty streak is due to an accumulation of lipid-laden foam cells in the intimal layer of the artery.

The results of the current study provide new insight into the role of the AHR and NLRP3 mediating the atherogenic effects of PM_2.5_ collected from traffic related air pollution (TRAP) in combination with nutritional factors of a HFD as illustrated in Figure 8. Combined data from mRNA expression analysis of CYP1A1, the DRE luciferase transactivation assay and GMSA clearly show the effectiveness of TRAP PM_2.5_ activating the AHR signaling pathway. This is supported by the recent literature indicating an important role of AHR mediating inflammatory responses of PM_2.5_, especially vehicular-specific PM collected in urban areas [20,21]. The current study shows that PM_2.5_ is a potent inducer of the inflammatory cytokines IL-1β, IL-8 and IL-33 in macrophages and IL-6 in HAEC. IL-1β and IL-8 including the receptor CXCR2 are critical players in atherogenesis and plaque disruption [23,31]. The role of IL-33 in atherogenesis is less clear and may include a protective function in CVD [32]. Further, treatment of Umac and HAEC with PM_2.5_ led to elevated mRNA levels of atherogenic markers COX-2 and PAI-2. Both genes have been described as AHR-responsive genes [33,34] and are rapidly activated by inflammatory stimuli [35,36]. The increased expression of COX–2 and PAI-2 in macrophages accumulated in atherosclerotic lesions have been found to promote the growth and rupture of plaques [37,38].

In addition, we tested nutritional factors since HFD and obesity are well known risk factors of atherosclerosis [6] and nutritional components such as glucose, cholesterol, and saturated free fatty acids may induce inflammatory cytokines [26]. Interestingly, high levels of glucose in the culture medium of macrophages further increased the PM-induced expression of COX-2 and PAI-2 as well as IL-1β, IL-8, and IL-33. A previous study also found an additional effect of glucose on PM-induced expression of IL-1β and IL-8 in Umac [39]. Dabir et al. [40] found that glucose activates AHR which was sufficient to induce the pro-atherogenic factor thrombospondin-1 (TSP-1) in endothelial cells. According to mechanistic studies with a TSP-1 promoter the authors concluded that glucose promoted the complex formation of AHR with Egr-1 and AP-2. Current results from CYP1A1 mRNA analysis and DRE luciferase reporter assays indicate that glucose and triglyceride support the PM_2.5_-mediated activation of the classical AHR/ARNT pathway. The induction and sustained increase in CYP1A1 activity can generate reactive oxygen species (ROS) contributing to stress and inflammation mediated by pollution and nutritional factors of a HFD [41]. In addition to chronic inflammation, elevated levels of ROS may further promote the development of obesity-associated disorders and atherosclerosis [42]. Furthermore, we found a significant increase in inflammatory cytokines, COX-2 and VEGF by palmitic acid but not by glucose in HAEC. Elevated levels of VEGF in endothelial cells may lead to leaky vessels and has been found to be associated with pathological conditions in atherosclerosis [43,44]. Interestingly, the expression of ANGPT was suppressed by TCDD, PM_2.5,_ and palmitic acid in HAEC. ANGPT is an angiogenic factor and may protect endothelial cells from pro-atherogenic effects of VEGF and inflammatory cytokines [45], thus its downregulation may further contribute to the development of atherosclerotic lesions. On the other hand, the PM_2.5_-induced expression of CRP and S100A9 was further elevated in the presence of cholesterol and palmitic acid but not by glucose or triglyceride in Umac. The acute phase protein CRP and the damage-associated molecular pattern molecule S100A9 play significant roles in atherogenesis and in plaque instability [5,46] and may also lead to the formation of foam cells via phagocytosis of low-density lipoprotein in macrophages [6,25,46]. Based on the significance of CRP and S100A9 in atherogenesis we investigated the pathways involved in the PM_2.5_-mediated regulation of the genes. The transcriptional activation of CRP and S100A9 by PM_2.5_ was found to be AHR-dependent which confirms previous studies showing the PM- and TCDD-mediated induction of CRP in macrophages and S100A9 in thymocytes and keratinocytes [7,47,48]. On the other hand, selective modulation of AHR activity has been found to suppress cytokine-induced CRP expression [49]. The additional effects of cholesterol and palmitic acid on the PM-induced CRP and S100A9 expression, however, was suppressed by NLRP3 silencing. Recent studies suggest that pattern recognition receptors such as NLRP3 play a critical role in detecting obesity factors including cholesterol and dietary fatty acids, which may initiate an inflammatory response [26,50,51]. The results suggest that simultaneous activation of the AHR and the NLRP3 by exposure to PM combined with cholesterol or palmitic acid enhances the expression of atherogenic markers. Furthermore, we found that a C/EBP binding element on the proximal promoter of CRP and S100A9 is required to mediate the PM_2.5_- and TCDD-induced activation of CRP and S100A9 which is supported by inhibition of C/EBP and PKA. The enzymatic activity of PKA has been described to be involved in transcriptional activation of C/EBPβ and its DNA binding [52]. A consensus DRE was detected at -3383 bp upstream of the start site of the S100A9 promoter [48], however, the CRP-proximal promoter and the S100A9 promoter constructs used in this study do not contain a consensus DRE element. The C/EBP binding sites close to the start site of CRP and S100A9 were found to bind C/EBPβ and mediate the transcriptional activation by inflammatory stimuli [53,54]. In previous studies the AHR-dependent and TCDD-induced expression of COX-2 and IL-1β have also been found to be mediated via PKA and enhanced DNA binding activity of C/EBPβ [52,55]. Together the current findings indicate that PKA and C/EBP are important components of an alternative AHR pathway in the regulation of inflammatory markers which are critically involved in chronic inflammatory diseases such as atherosclerosis. Furthermore, obesity factors including cholesterol and palmitic acid may interact with AHR signaling through the NLRP3 inflammasome and enhance the activation of markers of atherogenesis. Future studies will show if specific AhR antagonists or selective AhR modulators can provide a new therapeutic strategy to prevent CVD, especially for people exposed to high levels of air pollution.

## 4. Materials and Methods

### 4.1. Reagents and Preparation of PM

Dimethylsulfoxide (DMSO), phorbol-12-myristate-13-acetate (TPA), D-glucose, fructose, cholesterol, triglyceride, and palmitic acid were purchased from Sigma (Aldrich, St. Louis, MO, USA). [y-^32^P]ATP (6000 Ci/mmol) was purchased from ICN Biochemicals, Inc. (Costa Mesa, CA, USA). TCDD (>99% purity) was originally obtained from Dow Chemical Co. (Midland, MI, USA). The AHR antagonist 3′-methoxy-4′nitroflavone (MNF) was a kind gift of Josef Abel (IUF, University of Düsseldorf, Germany). The TRAP-related PM_2.5_ was collected from an exposure facility immediately adjacent to a major freeway tunnel system in Northern California [56] via impaction-based filter sampling and extracted according to the protocols of Bein and Wexler [57,58]. The dry PM extracts were resuspended in DMSO and sonicated immediately prior to injection in the cell cultures. Other molecular biological reagents were purchased from Qiagen (Valencia, CA, USA) and Roche Clinical Laboratories (Indianapolis, IN, USA). TCDD, MNF, PM extracts, and palmitic acid were prepared in DMSO as a 1000-fold concentrated stock solution. The final concentration of DMSO in the cell culture medium was 0.1% for single treatment. When cells were co-treated with TCDD, or PM plus MNF, or palmitic acid, the final DMSO concentration was 0.2%. Cholesterol was prepared in ethanol and the final concentration of the vehicle control was 0.1% ethanol. Fructose, glucose, and triglyceride were prepared in sterile water. The definitions of the abbreviations used in this article are listed in Abbreviations Section.

### 4.2. Cell Culture

The human monocytic cell line U937 was obtained from ATCC and maintained in RPMI 1640 medium. TPA (5 µg/mL) was used to differentiate the U937 into macrophages [7]. Bone marrow-derived macrophages (BMM) from mice were prepared as described [59]. AHR^−/−^ mice, kindly provided by Dr Christopher Bradfield from the McArdle Laboratory for Cancer Research at the University of Wisconsin, were genotyped using the DNA/RNA Shield™ kit (Zymo Research, Irvine, CA, USA). Human aortic endothelial cells (HAEC) kindly provided by Hnin Hnin Aung (UC Davis, CA, USA) were purchased from Lonza (Portland, OR, USA) and cultured in EBM basal media supplemented with EGM-2 bullet kit as described [9]. ]. The experiments with human cell lines were approved by the Biological Use Authorization (R1747) Committee of the University of California Davis (27 Nov 2017).

### 4.3. RNA Isolation and Quantitative Real-Time RT-PCR (qPCR)

Total RNA was isolated using a Quick RNA isolation kit (Zymo Research) and the cDNA was synthesized as described [60]. qPCR was then performed with the LightCycler LS480 (Roche, Indianapolis, IN, USA) using the Fast SYBR Green Master Mix (Applied Biosystems Inc., Foster City, CA, USA) according to the manufacturer’s protocol.

### 4.4. Transfection Experiments and Luciferase Assay

Transfection of plasmid DNA or small interfering RNA (siRNA) into Umac was performed via Nucleofector technology as described [61]. Briefly, Umac were resuspended in 100 μL Nucleofector Solution V (Amaxa GmbH, Köln, Germany) and nucleofected with 1.0 μg plasmid DNA or siRNA using program V-001, which is preprogrammed into the Nucleofector device (Amaxa GmbH). The CRP promoter constructs were cloned [53] and kindly provided by Alok Agrawal (East Tennessee State University, TN, USA). The S100A9 promoter constructs were generated as described [54,62] and kindly provided by Daigo Sumi (Tokushima Bunri University, Japan). The protein kinase A inhibitor expression vector (PKA-i) was kindly provided by Albert Smolenski (UCD Conway, Dublin, Ireland). The A-C/EBP vector was kindly provided by Charles Vinson (NCI, Bethesda, MD, USA) and produced dominant-negative proteins that specifically inhibit the DNA binding of the C/EBP members. The siRNA to target human AHR, NLRP3 and a negative control siRNA were synthesized by Qiagen. After transfection, Umac were incubated with the nutritional factors, TCDD, and PM_2.5_ for 16 h. Cells were lysed and luciferase activity was measured with the Luciferase Reporter Assay System (Promega Corp., Madison, WI, USA) using a luminometer (Berthold Lumat LB9501/16; Pittsburg, PA, USA). Relative light units were normalized to protein concentration using Bradford dye assay (Bio-Rad Laboratories, Inc., Hercules, CA, USA).

### 4.5. Gel-Mobility-Shift Assay (GMSA)

Nuclear extracts were isolated from Umac as described previously [61]. Protein-DNA complexes were resolved on a 4% non-denaturing polyacrylamide gel and visualized by exposure of the dried gels to X-ray films.

### 4.6. Statistics

The results were analyzed by GraphPad Prism software. All experiments were repeated a minimum of three times, and data are expressed as mean ± S.D. Differences were considered significant at *p* < 0.05. A comparison of two groups was made with an unpaired, two-tailed Student’s *t* test. A comparison of multiple groups was made with analysis of variance (ANOVA) followed by a Dunnett’s or Tukey’s test. A two-way ANOVA was used when data with more than one factor were analyzed.

## Abbreviations

AHR	Aryl hydrocarbon receptor
ANGPT	Angiopoietin
ARE	antioxidant response element
ARNT	AHR nuclear translocator
BMM	Bone marrow-derived macrophages
C/EBPβ	CCAAT/enhancer-binding protein beta
CCL	C-C motif chemokine ligand
CXCL	C-X-C motif chemokine ligand
Chol	Cholesterol
COX-2	Cyclooxygenase
CRP	C-reactive protein
CVD	Cardiovascular disease
CXCR	CXC-motive chemokine receptor
CYP DMSO DRE	Cytochrome P450 Dimethylsulfoxide Dioxin responsive element
Fruc	Fructose
Gluc	Glucose
GMSA	gel-mobility shift assay
HAEC	Human aortic endothelia cells
HFD	High-fat diet
IL	Interleukin
MNF	3-methoxy-4-nitroflavone
NF-kB	Nuclear factor kappa-light-chain-enhancer of activated B-cells
NLRP3	NLR family pyrin domain containing 3
PAHs	Polycyclic aromatic hydrocarbons
PAI-2	Plasminogen activator inhibitor 2
Palm	Palmitic acid
PKA	Protein kinase A
PM	Particulate matter
qPCR	Quantitative Real-Time RT-PCR
S100A9	Damage-associated molecular pattern molecule
siRNA	small interfering RNA
TCDD	2,3,7,8-tetrachlorodibenzo-p-dioxin
TGL	Triglyceride
TPA	phorbol-12-myristate-13-acetate
TRAP	Traffic related air pollution
TSP-1	Thrombospondin-1
Umac	U937 derived macrophages (human)
VEGF	Vascular endothelia growth factor
